# Mathematical Models of E-Antigen Mediated Immune Tolerance and Activation following Prenatal HBV Infection

**DOI:** 10.1371/journal.pone.0039591

**Published:** 2012-07-02

**Authors:** Stanca M. Ciupe, Sarah Hews

**Affiliations:** 1 Department of Mathematics, Virginia Tech, Blacksburg, Virginia, United States of America; 2 School of Natural Sciences, Hampshire College, Amherst, Massachusetts, United States of America; Albert Einstein College of Medicine, United States of America

## Abstract

We develop mathematical models for the role of hepatitis B e-antigen in creating immunological tolerance during hepatitis B virus infection and propose mechanisms for hepatitis B e-antigen clearance, subsequent emergence of a potent cellular immune response, and the effect of these on liver damage. We investigate the dynamics of virus-immune cells interactions, and derive parameter regimes that allow for viral persistence. We modify the model to account for mechanisms responsible for hepatitis B e-antigen loss, such as seroconversion and virus mutations that lead to emergence of cellular immune response to the mutant virus. Our models demonstrate that either seroconversion or mutations can induce immune activation and that instantaneous loss of e-antigen by either mechanism is associated with least liver damage and is therefore more beneficial for disease outcomes.

## Introduction

Infection with Hepatitis B virus (HBV) leads to asymptomatic self-limiting infections in most immunocompetent adult infections and chronic infections in perinatal, early childhood, and immuno-compromised adult infections [1]–[3]. Perinatal vertical transmission from mothers that have HBV e-antigen (HBeAg) in their serum is associated with high infectivity [4]. Successful clearance of HBV virus is believed to be immune mediated, with combined innate, cellular and humoral immune responses playing a role in disease outcome [5]–[8]. In contrast, perinatal chronic HBV infections are characterized by high HBV DNA in serum, the presence of hepatitis B e-antigen, and normal alanine aminotransferase (ALT) levels which indicate limited killing of infected liver cells by the immune system [9], [10]. The absence of liver disease in chronic patients is attributed to the immunoregulatory functions of HBeAg, which serves as a tolerogen by inactivating HBeAg-specific T-cells through clonal deletion, ignorance, and anergy [11]–[13].

Spontaneous HBeAg loss marks transition from immune tolerance to immune clearance phase and is considered a beneficial event for disease prognosis, especially when it occurs at an early age [14]. Two mechanisms of HBeAg loss have been proposed: HBeAg seroconversion through emergence of an anti-HBe antibody (HBeAb) [9], [14]–[16] and mutations in the core promoter or precore region of HBV genome that affect HBeAg production leading to emergence of predominant HBeAg-negative virus strains [17]–[19]. The immune activation phase is characterized by increased ALT levels, necrotic inflammatory activity, and loss of circulating HBeAg. These events are correlated with exacerbation of liver injury and risk of progressing to cirrhosis of the liver and to hepatocellular carcinoma [9], [18], [20]. Following HBeAg loss most patients enter an inactive phase where ALT levels are normal, HBV DNA is small, and there is minimal liver damage [9]. However, relapses in active HBV replication with HBeAg negative virus may arise, which may be correlated to initial age of HBeAg loss [14], [21]. These relapses are followed by ALT flares and moderate to severe liver damage [21].

The management of chronic HBV infection requires further understanding of the host-virus interactions leading to viral persistence. We aim to understand the role of HBeAg in creating immunological tolerance, the events leading to HBeAg clearance, subsequent emergence of potent cellular immune response, and the extent of liver damage. To provide insight into these mechanisms we develop mathematical models of cellular immune responses to a wild type HBeAg-positive virus and investigate interactions and parameter regimes that allow for viral persistence and eventual immune reactivation. Previous models have studied the dynamics of HBV clearance during acute infections [22]–[24], the decay profiles of HBV levels during drug therapy in chronic infections [25]–[28], and the roles of immune responses in HBV pathogenesis [29]–[31].

In this study we use mathematical models to investigate the mechanisms leading to establishment of chronic hepatitis B infection and aim to understand how the presence of circulating HBeAg creates immunological tolerance. We derive and analyze a mathematical model of e-antigen induced T-cell anergy in an HBeAg-positive HBV infection. We then expand the model to investigate long-term virus dynamics when e-antigen is lost and T-cells become activated as a result of e-antigen seroconversion or HBeAg-positive virus mutation. In the seroconversion scenario, we determine the antibody levels required to successfully restore effector function to anergic T-cells. A prediction of our model is that sudden loss of e-antigen through induction of high antibody levels is beneficial to the host as it reduces overall liver cell death. While seroconversion is considered an important stage in HBV clearance [14], the mechanism of seroconversion is unknown and it is difficult to predict when it will occur. Studies have found a correlation between seroconversion and core and precore mutations in HBeAg-positive virus [18]. We expand our model to account for mutations from HBeAg-positive to HBeAg-negative virus strains and investigate the composition of the overall virus population under different mutation regimes. We determine that intermediate mutation rates giving rise to mixed e-antigen positive and negative virus populations are associated with high levels of liver cell death, while complete loss of HBeAg-positive virus strains is associated with mild liver disease and low HBeAg-negative virus levels, corresponding to inactive stages of HBV infection [9]. These results provide a clearer picture of the long-term hepatitis B virus dynamics and of the extent of liver disease following immune activation.

## Models and Methods

### Model of HBeAg mediated chronic HBV infection

Let 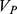 be HBeAg-positive virus concentration, 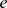 be HBeAg concentration and 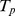 be the concentration of HBeAg-specific T-cells. Following infection with the virus, liver cells start producing new virions. We simplify the viral life-cycle, aggregating the processes of infection and host-cell viral production into a simple replication model in which viruses divide with a per-capita rate, 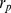, and a maximum carrying capacity, 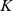. 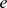 is produced at rate 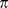 per virus per day and is cleared at rate 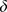 per day. 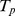 are activated upon encounter with antigen, clonally expand and differentiate into cytotoxic killer cells at rate 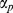, and have an average lifespan of 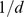 days. We model immunological tolerance by inhibiting the T-cell expansion by a maximum quantity 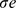. Finally, virus is removed by immune cells at rate 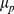. As immune cells do not kill virus directly, we assume that virus is in quasi-equilibrium with infected liver cells. The dynamics of the model are given by the following system:
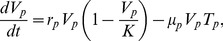
(1)

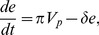
(2)

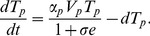
(3)


System (1–3) has three steady states: a biologically irrelevant steady state, 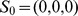, a state representing immune tolerance, 
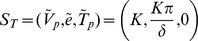
, and a state representing immune activation, 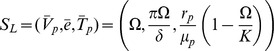; where 
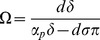
. These states correspond to liver failure, immune-tolerant chronic infection and immune-competent chronic infection respectively.


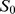
 is always unstable. If
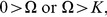
(4)



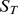
 is asymptotically stable and 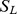 does not exist. If 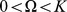 then 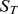 is unstable and 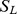 exists and is asymptotically stable. From here on we will refer to (4) as the tolerance conditions.

### Model for loss of HBeAg due to seroconversion

Several papers have documented the emergence of an anti-HBe antibody that binds e-antigen and enhances its removal [9], [14]–[16]. We model loss of e-antigen due to HBeAg-specific antibodies by increasing HBeAg clearance rate in equation (2) to account for antibody-mediated removal:
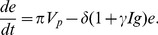
(5)



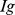
 represents HBeAb concentration, which we assume constant and equal to its maximal size and 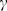 is the antibody-mediated HBeAg removal rate. The modified system still has the biologically irrelevant steady state, 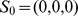, and the immune tolerance steady state, 
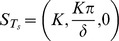
. The immune activation steady state becomes 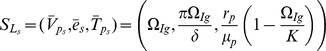, where 
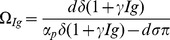
. Notice that 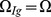 when 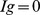.


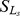
 exists and is asymptotically stable when 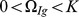. Following seroconversion, tolerance, represented by (4), is lost when enough HBeAg-specific antibody is present to make 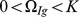.

### Model for loss of HBeAg-positive virus through mutations

Mutations in the hepatitis B virus core promoter region [32] or precore region [33] may affect HBeAg production leading to the emergence of HBeAg-negative virus strains and subsequent activation of T-cell clones that are specific for such virus [17]. We model this process by expanding system (1–3) to account for the emergence of HBeAg-negative virus concentration, 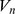, and the corresponding T-cell response, 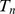. We assume that starting at time 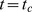 a continuous percent 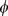 of the reproducing virus strain 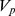 mutates into virus 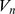. The T-cell response to the HBeAg-negative virus arises at 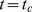 as well. The new system is given by:
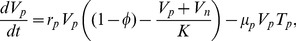
(6)

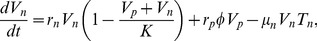
(7)

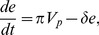
(8)

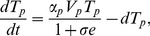
(9)

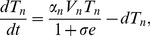
(10)where the dynamics of 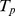 and 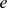 are as before. Viruses of strain 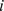 are produced at rates 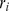 and removed at rates 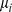 by their strain-specific T-cell responses (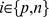). Upon encountering HBeAg-negative virus, 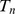 expands at rate 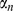 and is inhibited by the e-antigen by a maximum quantity 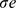. Their average lifespan is 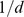 days. For simplicity we assume that the two viruses have the same fitness rates 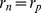 and the same removal by immune system rates 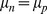.

The mutation model has several steady states. The first one is biologically irrelevant, 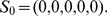 The tolerance state of (6–10) is depicted by the absence of T-cell induced killing of 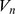 when 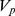 is lost completely, 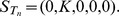


There are four steady states that represent immune activation. The first one represents immune activation against 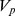 but not 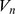, 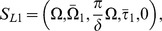 where 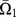 and 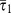 are defined in Supporting Information S1. 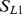 exists when 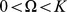 and 
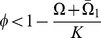
.

There are two steady states representing competent T-cell response to 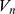 but not 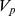: one corresponding to small and intermediate percentage of 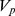 mutations, 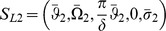 which exists and is asymptotically stable when 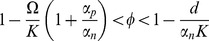 (
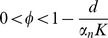
 when tolerance condition (4) is satisfied); and one corresponding to large percentage of mutations leading to complete removal of 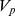, 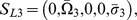 which exists when 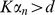 and is asymptotically stable when 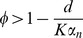.

The last steady state corresponds to T-cells response to both viruses types, 
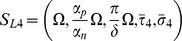
 which does not exist when 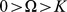. 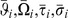 (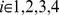) are defined in Supporting Information S1.

Under the tolerance condition (4) the only steady states that exist and can be stable are 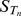, 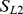 and 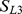 (for detailed analysis see Supporting Information S1).

### Initial data and immune tolerance

For model (1–3) we assume that the initial inoculum contains small concentrations of HBeAg-positive virus and circulating HBeAg (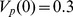 per ml, 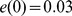 per ml). In the absence of infection the concentration of HBeAg-specific T-cells is 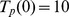 cells per ml.

For model (6–10), we assume initial concentrations of 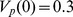 virus per ml, 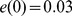 per ml, and 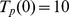 cells per ml, as in the initial model (1–3). Moreover, HBeAg-negative virus and corresponding T-cells are absent, *i.e*. 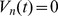, 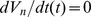, 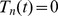, and 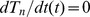 for 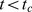. Therefore, for 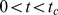, the dynamics of (6–10) are identical to the dynamics of (1–3), with the immune tolerant state 
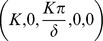
 being asymptotically stable when tolerance conditions (4) are satisfied. At 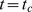 mutations start. We model this by the emergence of positive concentrations of 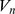 and 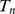 in the body, *i.e*. 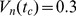 per ml and 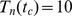 cells per ml (similar dynamics will be achieved if 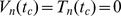). The rest of the variables are computed by solving (6–10) at time 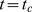. We study the conditions needed for the loss of tolerance under this scenario.

## Results

### Immune tolerance

The presence of circulatory HBeAg can induce clonal anergy characterized by the inability of HBeAg-specific T-cells to grow, maturate, and acquire effector function such as cytokine production [11]–[13]. Analytical results show that this is represented by parameters of model (1–3) satisfying conditions (4). We rewrite them to study the effect of the HBeAg:
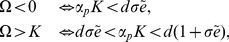
(11)where 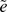 is the steady state concentration of e-antigen during immune tolerance. Biologically, our model predicts that when the maximal activation of the immune cells is smaller than immune cells removal rate at the peak of e-antigen inhibition or smaller than the combined effect of natural death rate and removal rate at the peak of e-antigen inhibition then the immune cells are lost. The virus replicates and settles at high levels of 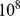 HBV copies per ml. Inequality (11) provides an estimate for the e-antigen levels required for induction of T-cell tolerance.

An extreme case describing this scenario is represented by the failure of T-cells to differentiate, 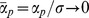 (in particular 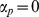) and would correspond to HBeAg-specific T-cell ignorance. This is in agreement with experimental observations [11]. Under the 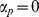 assumption, system (1–3) has only two steady states: 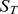, which is always stable, and 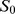, which is always unstable.

### Increased CTL production leads to loss of tolerance

Since the loss of tolerance is marked by an increase in HBeAg-specific T-cell levels, we first explore the causes of this increase. In our model, immune recovery is represented by change in stability from the tolerance steady state 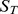 to the immune activation steady state 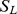. This can occur when either the differentiating rate of HBeAg-specific T-cells (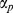) increases or the inhibition of differentiation due to HBeAg (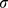) decreases causing
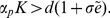
(12)


Biologically, this means that when the maximal activation of HBeAg-specific T-cells is bigger than the combined effect of their natural death rate and the death rate at the peak of e-antigen inhibition then the tolerance is lost and the immune cells start removing virus. Condition (12) is independent of the size of the immune cells killing rate (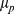), which means that the presence of HBeAg does not affect directly the killing capacity of HBeAg-specific T-cells.

**Figure 1:**
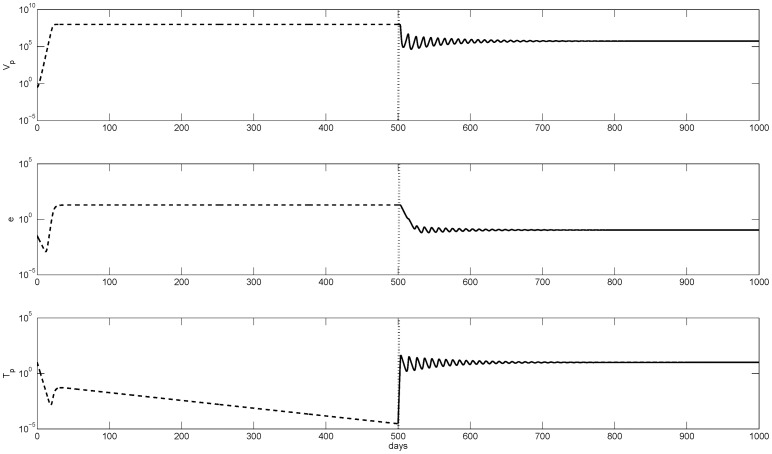
Numerical curves given by model (1–3) showing the temporal evolution from tolerance (dashed lines) to immune activation (solid lines) when the activation parameter 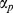 is increased from 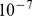 to 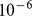. The transition point is marked by vertical lines. The other parameters are as listed in table 1.

The temporal transition from immune tolerance to immune activation is presented in figure 1. High HBeAg-positive virus concentration of 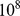 copies per ml decrease to below 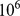 copies per ml (see figure 1 top panel) when the ignorant HBeAg-specific T-cells (lower panel, dashed line) get activated (lower panel, solid line). The loss of tolerance is marked by vertical lines.

Notice that (12) also suggests that tolerance can be lost due to a net decrease in the HBeAg production. To place this on solid biological ground, we consider HBeAg seroconversion.

### Loss of HBeAg due to seroconversion leads to immune activation

**Figure 2:**
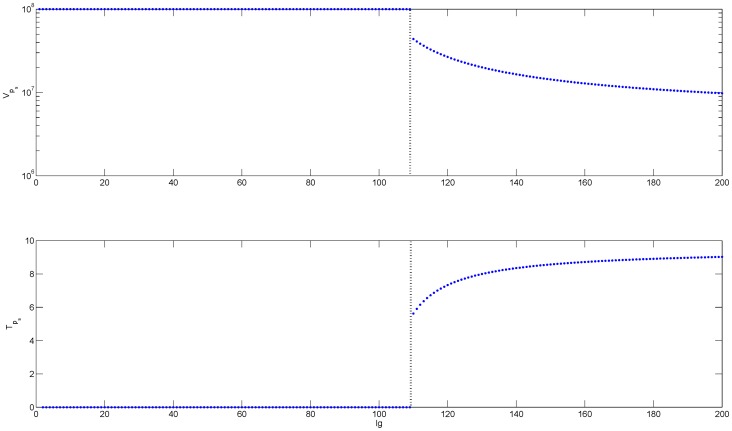
Bifurcation diagrams showing the change in the steady states of HBeAg-positive virus (top panel) and HBeAg-specific T-cells (lower panel) as a function of antibody levels (Ig) for 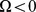. We used the parameter values listed in table 1, 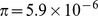 and 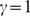. The values for 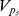 and 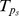 are given by steady state values 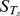 for 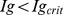 and 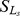 for 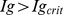, and the transition from tolerance to immune activation at 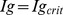 is marked by vertical lines.

Analytical investigation of model (1–3) with e-antigen equation modified to (5) states that when 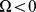 and 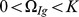, we observe immune tolerance for small antibody levels (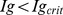) and immune activation and killing in the presence of potent antibody response (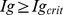) (see figures 2 and S1). Note that a high antibody removal rate is necessary for the HBeAg-specific T-cells to reach steady-state values. When 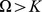 and 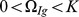 we observe immediate immune activation and killing even for low antibody levels, *i.e*. 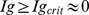.

For both cases (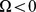 or 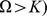, the critical antibody response needed for HBeAg-specific T-cells to reach equilibrium levels is given by
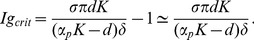
(13)


It should be mentioned that 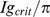 is the same for 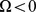 or 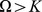. Since 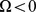 corresponds to higher levels of HBeAg production 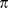, higher antibody levels are needed for immune activation to occur, *i.e* 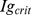 is bigger in the 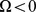 than in the 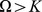.

**Figure 3:**
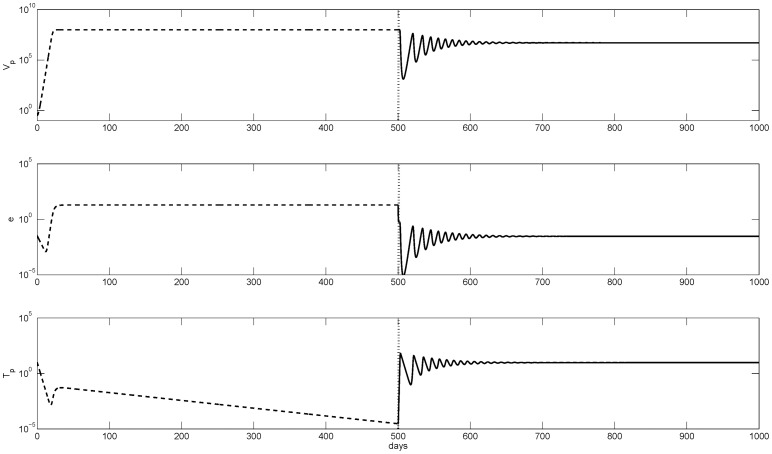
Numerical curves showing the temporal evolution from tolerance given by model (1–3) (dashed lines) to immune activation due to seroconversion given by model (1-5-3) (solid lines). The transition point is marked by vertical lines. The parameters are as in table 1, 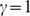, and 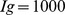.

Biologically, immune tolerance occurs when maximal activation of HBeAg-specific T-cell is lower than the combined effect of their natural death rate and death rate at the peak of HBeAg inhibition. Immune tolerance is lost when maximal activation of HBeAg-specific T-cell exceeds the combined effect of their natural death rate and death rate at the peak of HBeAg inhibition in the presence of HBeAg-specific antibodies. Temporal evolution of the HBeAg-positive virus, HBeAg, and HBeAg-specific T-cell concentrations prior and following seroconversion are presented in figure 3. We see that when antibodies bind HBeAg (vertical lines), hepatitis B virus decays from 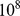 virions per ml (top panel, dashed line) to 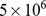 virions per ml (top panel, solid line). At the same time, low HBeAg-specific T-cell levels seen before seroconversion (bottom panel, dashed line) start increasing to positive steady state values following removal of HBeAg (bottom panel, solid line).

### Loss of HBeAg-positive virus through mutations

Mutations in the hepatitis B virus core promoter region [32] or precore region [33] may affect HBeAg production leading to the emergence of HBeAg-negative virus strains and subsequent activation of T-cell clones that are specific for such virus [17]. We used the two virus model presented in the methods section to study this possibility.

Analytical results show that there are three states that exist and can be stable under the tolerance condition (4): immune tolerance to virus 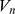 when 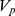 completely mutates (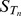), competent T-cell response to 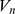 but not 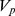 when small and intermediate percentage of 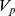 mutates (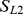), and immune activation and complete loss of 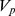 (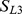).

Analytical results show that the immune response to the mutant virus, 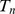, cannot persist and the tolerance is not lost when the T-cell activation rate at the carrying capacity of the virus is smaller than the T-cell death rate (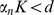). Virus population consists exclusively of HBeAg-negative virus who reached its carrying capacity (see figure S2).

**Figure 4:**
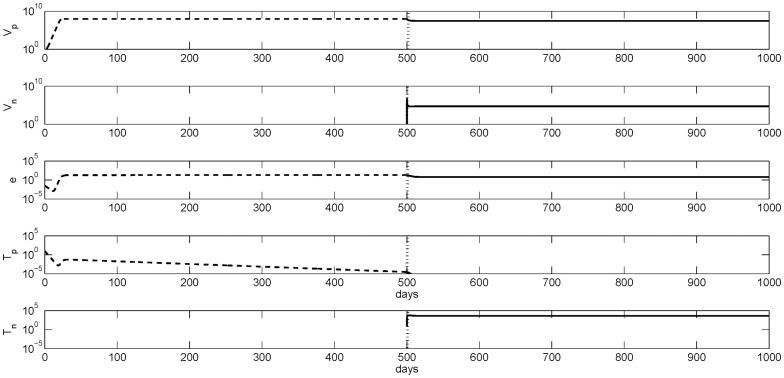
Numerical curves showing the transition from an immune tolerant HBeAg-positive virus 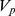 virus given by 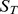 stable in model (1–3) (dash lines) to an HBeAg-negative virus mediated immune activation state under small and intermediate mutation rates corresponding to 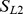 stable in model (6–10) (solid lines). The transition is marked by vertical lines. Parameters are 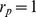, 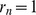, 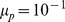, 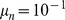, 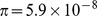, 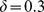, 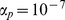, 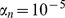, 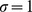, 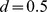, 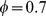 and 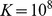. The tolerance steady states of the one-virus model are used as initial conditions for the two virus model with 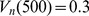 and 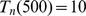.

When the activation rate at the carrying capacity of the virus is larger than the T-cell death rate (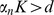), the tolerance state 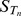 becomes unstable and the immune response to the mutant virus, 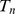, reaches a positive concentration. Analytical results predict that, depending on several factors, partial or complete loss of HBeAg occurs. When the replication rate of the HBeAg-positive virus exceeds the virus loss due to competition with HBeAg-negative virus, 
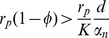
, the two viruses will coexist (see figure 4) and 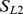 is stable. The overall virus population st steady state is 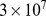 HBV per ml (first two panels, solid lines) compared to the 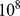 HBeAg-positive HBV per ml in the tolerant stage (top panel, dashed line). There is no HBeAg-specific T-cell response (forth panel, solid line), but a potent T-cell response to HBeAg-negative virus emerges (bottom panel, solid line).

**Figure 5:**
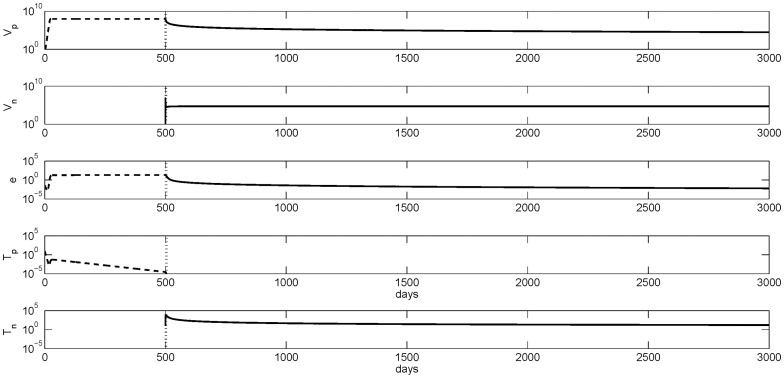
Numerical curves showing the transition from an immune tolerant HBeAg-positive virus 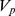 virus given by 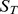 stable in model (1–3) (dash lines) to an HBeAg-negative virus mediated immune activation state under large mutation rates corresponding to 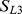 stable in model (6–10) (solid lines). The transition is marked by vertical lines. Parameters are as in figure 4 and 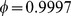. The tolerance steady states of the one-virus model are used as initial conditions for the two virus model with 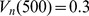 and 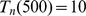.

Conversely, when the replication rate of the HBeAg-positive virus is smaller than virus loss due to competition with the HBeAg-negative virus, 
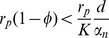
, then, at steady state, the HBeAg-positive virus is eliminated (see figure 5, top panel, solid line), 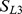 becomes stable, and the emerging HBeAg-negative virus reaches a low steady state of 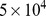 HBV per ml (see figure 5, second panel, solid line). This corresponds to an inactive carrier state [9]. There is no T-cell response to the HBeAg-positive virus (forth panel, solid line), but a potent T-cell response to HBeAg-negative virus emerges (bottom panel, solid line).

### Liver Damage due to Seroconversion

Following seroconversion with critical antibody levels, both HBeAg and HBeAg-positive virus decrease but are not completely eliminated. At the same time, HBeAg-specific T-cells are activated and start killing the virus. Transition from immune ignorance to immune activation is almost instantaneous and accounts for the loss of more than 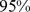 of the virus (under parameter values presented in table 1). To determine the extent of liver injury corresponding to virus loss following seroconversion we assume that the infected cells (hepatocytes), 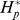, are in quasi-equilibrium with the virus:
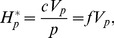
(14)where 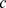 is the virus clearance rate per day, 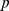 is the number of viruses produced by an infected cell per day, and 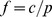 is the number of infected cells per virus.

During the tolerance phase, 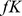 hepatocytes per ml are infected at steady state. Following seroconversion, this amount is reduced and, at steady state, it reaches 
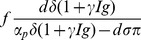
 hepatocytes per ml. For parameters values listed in table 1, 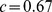 per day and 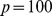 viruses per infected cell per day [30], we estimate that there are 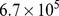 infected hepatocytes per ml at steady state during immune tolerance and 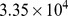 infected hepatocytes per ml at steady state during immune activation. This corresponds to a loss of less than 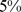 of the liver (where the liver size is estimated as containing 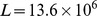 hepatocytes per ml).

Since the immune activation does not lead to viral eradication, the HBeAg-specific T-cells will continue to kill liver cells over time. The cumulative hepatocyte loss on the first 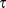 days is given by
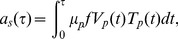
(15)where 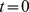 is the time when antibody levels become positive, 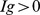. As the immune cell population 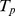 is small (but non-zero), the cumulative hepatocyte loss 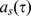 is nonzero as well for solutions of system (1-5-3) with 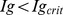. However, we observe significant liver damage only after 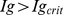.

**Figure 6:**
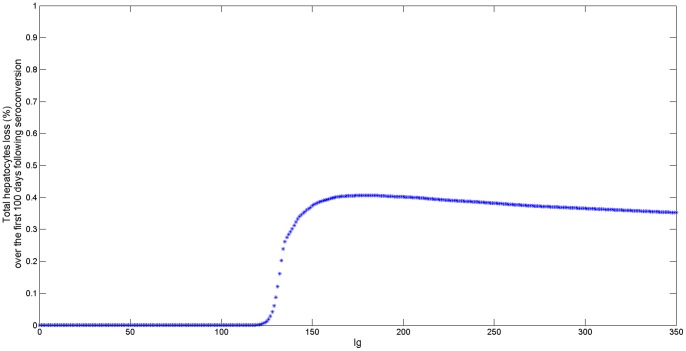
Cumulative % liver loss as a function of antibody 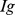 for 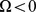. The liver loss is computed as 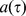/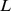 where 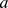 is given by (15), 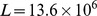 represents the total hepatocytes concentration in the liver and 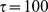. The rest of parameters are given in table 1.

As expected, the amount of liver damage will increase with the time spent in the activation stage. Numerical simulations of the relationship between liver damage and fixed antibody levels (figure 6) show that intermediate antibody levels accounts for the strongest hepatocyte removal. Cumulative hepatocytes loss saturates to lower values as antibody concentration increases. Therefore, induction of high antibody levels is more beneficial for the patient as it leads to less liver damage.

**Table 1:** Variable and parameter values used for simulations.

*Variable*	*Description*	*Units*	
V_p_	e-antigen positive HBV	ml^−1^	
V_n_	e-antigen negative HBV	ml^−1^	
e	e-antigen	ml^−1^	
T_p_	T-cells specific for e-antigen positive HBV	ml^−1^	
T_n_	T-cells specific foe e-antigen negative HBV	ml^−1^	
I_g_	e-antigen specific antibody	ml^−1^	

### Liver Damage due to Mutations

During mutation from HBeAg-positive into HBeAg-negative virus, tolerance is lost when the activation rate of T-cells specific for HBeAg-negative virus at the carrying capacity of the virus is bigger that the T-cell death rate. This is true for all mutation percentages and independent of our starting point 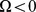 or 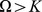.

**Figure 7:**
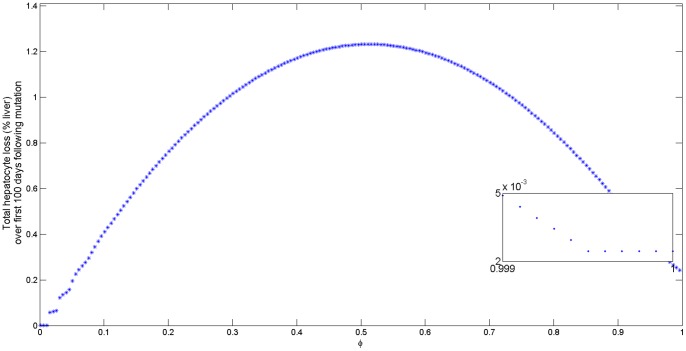
Cumulative % liver loss as a function of mutation rate 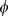 for 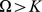. The liver loss is computed as 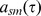/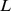 where 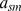 is given by (16). The bottom figure is computed as 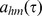/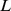 where 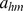 is given by (17) for 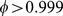. 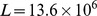 represents the total hepatocytes density in the liver, and 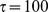. The rest of parameters are given in table 1.

For low and intermediate mutation rates, virus 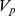 is still present and mutates continuously into 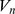. The cumulative hepatocyte loss on the first 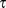 days is
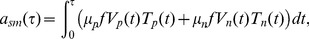
(16)where 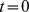 is the time of immune activation due to mutation. The maximum hepatocyte loss occurs for intermediate mutation rates (see figure 7 top panel).

For high mutations rates, the HBeAg-positive virus is lost at a slow rate. Many years of continuous mutation at high rate are needed before HBeAg-positive virus is completely eliminated. When HBeAg-positive virus is completely replaced by HBeAg-negative virus, the mutant virus persists at low levels 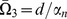. The cumulative hepatocyte loss on the next 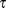 days following HBeAg-positive virus elimination is
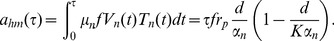
(17)


The overall hepatocyte death when only 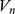 is present is small compared to the overall hepatocyte death when the virus population still contains 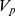 (see figure 7 bottom panel).

Our model predicts that the liver loss is compensated by the proliferation of hepatocytes,
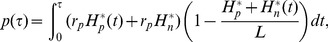
(18)where 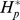 and 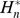 are hepatocytes infected with the e-antigen positive and negative virus strains. In spite of this compensation, the rapid liver cell turnover can lead to accumulation of mutations in the host genome that could result in genetic alterations, chromosomal rearrangements, activation of oncogenes, inactivation of tumor suppressor genes, and ultimately to hepatocellular carcinoma as seen in many patients with chronic hepatitis [34].

## Discussion

We formulated a model of immune tolerance during chronic infection with hepatitis B virus. The model considers the interaction between HBeAg-positive hepatitis B virus, HBeAg, and HBeAg-specific T-cells. We derived conditions under which pressure from HBeAg leads to failure of HBeAg-specific T-cells to efficiently expand and control the infection and investigated whether loss of HBeAg can lead to immune activation, virus removal, and eventual liver damage. We modified the model to account for two possible biological scenarios of HBeAg loss: the sudden production of HBeAg specific antibodies, and the mutation from HBeAg-positive to HBeAg-negative hepatitis B virus strains.

Our models demonstrate that either seroconversion or mutations can cause loss of tolerance. In the seroconversion model, immune tolerance is lost when 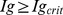, where 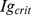 is proportional to the HBeAg production rate. When high levels of e-antigen are produced every day, HBeAg-specific T-cell response is so weak that its activation at the virus carrying capacity is smaller than the immune cells death rate (Ω < 0). In this case, large levels of HBeAg-specific antibodies are needed for immune activation to occur. When lower levels of e-antigen are produced every day, the HBeAg-specific T-cell activation rate at the virus carrying capacity is greater than the death rate but smaller than the combined effect of their natural death rate and death rate at the peak of HBeAg inhibition (Ω > K), small levels of HBeAg-specific antibodies are needed for immune activation to occur. We predict increased liver damage when the minimum amount of antibody needed for immune activation is present (with the highest damage being done for the 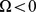 parameter space) and decreased liver loss when high antibody levels are reached. This implies a more beneficial outcome in the disease prognosis if the seroconversion is instantaneous rather then gradual. This result is in agreement with biological studies that show that gradual loss of HBeAg-positive virus through intermediate mutations [14] or through persistence of HBeAg-positive virus strains through integration into hepatocyte genome [19] can harm the patient.

In the mutation model, immune tolerance is lost when the activation rate of T-cells specific for HBeAg-negative virus at the carrying capacity of the virus is larger than their death rate. Furthermore, the removal of HBeAg-positive virus and HBeAg and subsequent liver damage is driven by the mutation rate. The highest liver cell loss takes place when half of the HBeAg-positive virus mutates. If more than 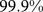 hepatocytes mutate over a long period of time, then the liver damage is insignificant. This implies that instantaneous loss of HBeAg through mutation is beneficial to the disease outcome leading to inactive carrier states as previously suggested [19]. Our mutation model makes three assumptions: (1) that the percent 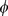 of strain 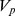 that mutates to give rise to virus 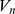 is constant; (2) that there is no cross-reactivity between the T-cell response to HBeAg-negative virus and the HBeAg-positive virus, and (3) that the dynamics of hepatocytes infection and viral production can be incorporated into a single virus equation. Based on the asymptotic analysis of (6–10), varying 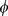 does not affect the stability of the tolerance state 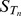. It does affect whether e-positive virus will coexist with e-negative virus and HBeAg would be present or e-positive virus and e-antigen would be lost. Therefore, relaxing the first assumption is unlikely to dramatically affect our conclusions. The second assumption can be addressed by modifying the mutation model to account for T-cells cross-reactivity. If we assume that HBeAg-positive and HBeAg-negative virus replicate at the same rates and cross-reactive T-cells remove HBeAg-positive virus at a higher rate than HBeAg-negative virus (a phenomenon known as original antigenic sin [35]), then HBeAg-positive virus is removed completely, regardless of mutation rate when the cross-reactive T-cell activation rate at the virus carrying capacity is greater than its death rate. In this case, the patient enters an inactive carrier stage where liver damage is minimal. The third assumption can be addressed by modifying the mutation model to account for the dynamics of uninfected and infected hepatocytes. One important biological assumption is that unlike acute HBV infection, where the majority of the liver gets infected by HBV, chronic HBV infection leads to a smaller percentage of infected hepatocytes [36], [37]. Because of this, the HBeAg-negative virus has enough target cells to infect, and following continuous mutation, to become the dominant virus in the body. We compared the stability results for the extended model with the results predicted by the mutation model presented in the paper and found that increasing model complexity does not change the results (not shown). However, while we still obtain high liver damage for intermediary mutation rates, the peak of liver loss shifted to the right and occurs for 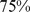 rather than 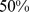 mutation rate (not shown). This result may be due to the fact that delay in hepatocyte infection and T lymphocytes specific for HBeAg-negative virus activation may be accounted by the extended model. Further analysis is needed to validate these conjectures.

Our model predicts that for small mutation rates the level of HBeAg-positive virus is 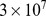 copies per ml. If we take into account the hypothesis that loss of HBeAg through mutations in the core promoter can cause HBeAg seroconversion [18], and transform the mutation model to account for e-antigen loss at rate 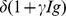 with 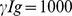 being the overall HBeAg removal by antibody, then the HBeAg-positive virus (which accounts for the majority of HBV DNA) decreases by one order magnitude to 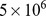 copies per ml. However, the overall dynamics and quantity of the liver loss remains unchanged, with high levels of liver cell death for intermediate mutation rates.

An interesting implication of this model is that tolerance and loss of tolerance are not affected by the virus replication rate (*r_p_*) and T-cells killing rate (*μ_p_*), as the size of 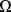 does not depend on these parameters. The independence of tolerance loss on T-cell killing rate is preserved in both seroconversion and mutation models. This suggests that the e-antigen induces T-cell tolerance by reducing their proliferative but not their killing capabilities.

Our model does not consider all host and viral mechanisms associated with hepatitis B virus tolerance. While we assumed that T-cells inactivity is solely mediated by e-antigen, it has been suggested that the core antigen (HBcAg) may act as a tolerogen as well [11]. Furthermore, recent studies have shown that the proliferative capacities of CD8 T-cells during chronic hepatitis are affected by increased regulatory T-cell (Tregs) levels [38], [39] and by an imbalance between Tregs and T helper cells that produce interleukin-17 (Th17) [40]. Further work is needed to account for the quantitative contributions of each of these factors. Finally, we ignored the age of tolerance loss, which may provide insight into the correlation between the age of the patient at immune activation and the severity of infection [14], [21]. In spite of these simplifications, our study makes predictions on the type of dynamics expected during tolerance loss. Such understanding is essential in determining whether its occurrence is desirable and whether we can control immune activation so as to minimize long term negative effects on the patient's liver.

## Supporting Information

Figure S1
**Bifurcation diagrams showing the change in the HBeAg-positive virus steady state (top panel) and HBeAg-specific T cells steady state (lower panel) as a function of antibody levels Ig for 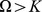.** We used parameters from table 1, 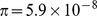 and 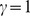. The transition from tolerance to immune activation is marked by vertical lines.(TIF)

Figure S2
**Numerical curves showing the transition from an immune tolerant HBeAg-positive virus 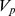 virus given by 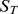 stable in model (1–3) (dash lines) to an immune tolerant mutant HBeAg-negative virus 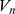 virus given by 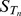 stable in model (6–10) (solid lines).** The transition is marked by vertical lines. Parameters are 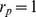, 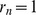, 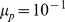, 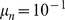, 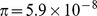, 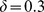, 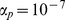, 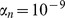, 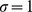, 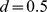, 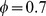 and 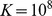. The tolerance steady states of the one-virus model are used as initial conditions for the two virus model with 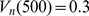 and 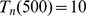.(TIFF)

Supporting Information S1 Here we perform asymptotic analysis for the models given by (1–3) and (6–10).
(PDF)
