# Supplementary figures and images for: Mathematical Models of E-Antigen Mediated Immune Tolerance and Activation following Prenatal HBV Infection
Source: PLoS One. 2012 Jul 2;7(7):e39591. doi: 10.1371/journal.pone.0039591 (PMC3388102; doi:10.1371/journal.pone.0039591)

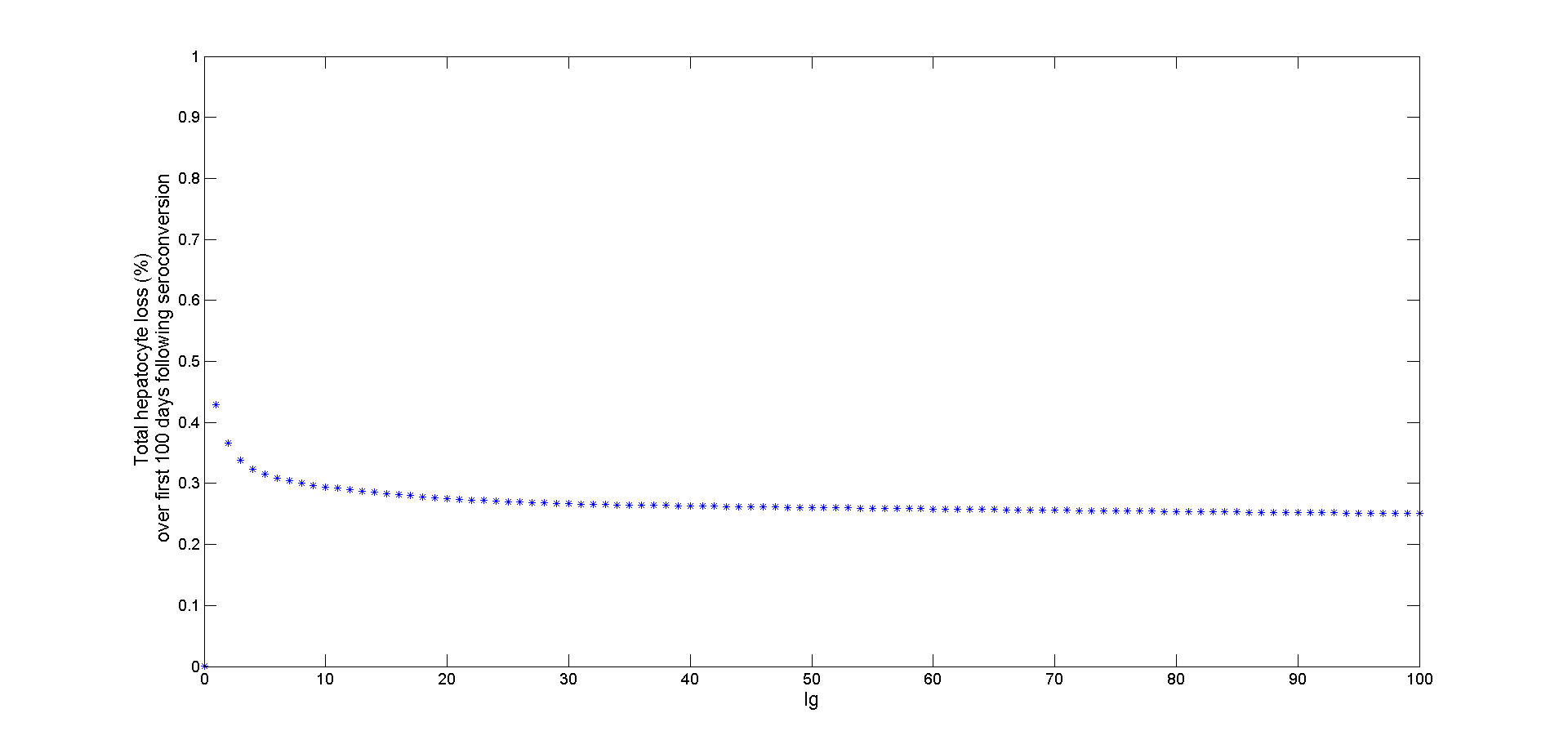

Supplement: Figure S1 — Bifurcation diagrams showing the change in the HBeAg-positive virus steady state (top panel) and HBeAg-specific T cells steady state (lower panel) as a function of antibody levels Ig for . We used parameters from table 1, and . The transition from tolerance to immune activation is marked by vertical lines. (TIF) [file pone.0039591.s001.tif]
