# Supplementary material for: Mathematical Models of E-Antigen Mediated Immune Tolerance and Activation following Prenatal HBV Infection
Source: PLoS One. 2012 Jul 2;7(7):e39591. doi: 10.1371/journal.pone.0039591 (PMC3388102; doi:10.1371/journal.pone.0039591)
Supplement: Supporting Information S1 — Here we perform asymptotic analysis for the models given by (1–3) and (6–10). (PDF) [file pone.0039591.s003.pdf]

Mathematical models of immunological tolerance and immune activation following prenatal infection with hepatitis B virus Supplementary Materials

Model analysis of one-virus model

$$\frac{dV_p}{dt} = r_p V_p \left(1 - \frac{V_p}{K}\right) - \mu_p V_p T_p, \quad (1)$$

$$\frac{de}{dt} = \pi V_p - \delta e, \quad (2)$$

$$\frac{dT_p}{dt} = \frac{\alpha_p V_p T_p}{1 + \sigma e} - d T_p. \quad (3)$$

Proposition 1. *Each component of the solution of system (1-3), subject to* $0 \leq V_p(0) \leq K$, $e(0) \geq 0$, $T_p(0) \geq 0$ *remains bounded and non-negative for all* $t > 0$.

Proof. Because the system (1-3) is locally Lipschitz at $t = 0$, its solution exists on the interval $[0, b)$ for some number $b > 0$.

Suppose that there exists $t_1 \in (0, b)$ such that $e(t_1) = 0$, and $V_p(t) > 0$, $e(t) > 0$, $T_p(t) > 0$ for $0 < t < t_1$. For all $t \in [0, t_1]$ we have

$$\frac{de}{dt} = \pi V_p - \delta e \geq -\delta e.$$

The exponential solution is a lower bound of $e(t)$, and $e(t_1) \geq e(0)e^{-\delta t_1} > 0$, which contradicts the $e(t_1) = 0$ assumption.

Similarly, assume there exists $t_1 \in (0, b)$ such that $T_p(t_1) = 0$, and $V_p(t) > 0$, $e(t) > 0$, $T_p(t) > 0$ for $0 < t < t_1$. For all $t \in [0, t_1]$ we have

$$\frac{dT_p}{dt} = \frac{\alpha_p V_p T_p}{1 + \sigma e} - d T_p \geq -d T_p.$$

The exponential solution is a lower bound of $T_p(t)$, and $T_p(t_1) \geq T_p(0)e^{-dt_1} > 0$, which contradicts the $T_p(t_1) = 0$ assumption.

Finally, assume there exists $t_1 \in (0, b)$ such that $V_p(t_1) = 0$, and $V_p(t) > 0$, $e(t) > 0$, $T_p(t) > 0$ for $0 < t < t_1$. For all $t \in [0, t_1]$ we have

$$\frac{dV_p}{dt} = r_p V_p \left(1 - \frac{V_p}{K}\right) - \mu_p T_p V_p \geq -\mu_p T_p V_p.$$

Then $V_p(t_1) \geq V_p(0)e^{-\mu \int_0^{t_1} T_p(t)dt} > 0$, which contradicts the $V_p(t_1) = 0$ assumption.

We can now verify that the solutions are bounded. Since

$$\frac{dV_p}{dt} = r_p V_p \left(1 - \frac{V_p}{K}\right) - \mu_p T_p V_p \leq r_p V_p \left(1 - \frac{V_p}{K}\right),$$

we have that V_p is bounded above by a solution to the logistic equation, with initial condition $V_p(0) \leq K$. We conclude that $V_p(t) \leq K$ for all $t \in [0, b)$.

Since $V_p(t) \le K$,

$$\frac{de}{dt} = \pi V_p - \delta e \le \pi K - \delta e$$

and $e(t) \le \frac{K\pi}{\delta} + \left(e(0) - \frac{K\pi}{\delta}\right) e^{-\delta t} \le \max\{e(0), \frac{K\pi}{\delta}\}$ for $t \ge 0$.

Finally, let $z = \alpha_p V_p + \mu_p T_p$. For $V_p(t) \le K$ and $e(t) \ge 0$,

$$\frac{dz}{dt} = \frac{\alpha_p \mu_p V_p T_p}{1+\sigma e} - \alpha_p \mu_p V_p T_p + r_p \alpha_p V_p \left(1 - \frac{V_p}{K}\right) - d\mu_p T_p \le (r_p + d)\alpha_p K - dz.$$

Therefore $z(t) \le \frac{(r_p+d)\alpha_p}{d} K + \left(z(0) - \frac{(r_p+d)\alpha_p}{d} K\right) e^{-dt} \le \max\{z(0), \frac{(r_p+d)\alpha_p}{d} K\}$. Since $V_p(t)$ and $z(t)$ are bounded it follows that $T_p(t)$ is bounded on $[0, b)$.

System (1-3) together with initial conditions $0 \le V_p(0) \le K$, $e(0) \ge 0$, $T_p(0) \ge 0$ has positive and bounded solutions for all $t \in [0, b)$. This implies that $b = \infty$. □

System (1-3) has three steady states: a biologically irrelevant steady state, $S_0 = (0,0,0)$, a state representing immune tolerance, $S_T = (\tilde{V}_p, \tilde{e}, \tilde{T}_p) = \left(K, \frac{K\pi}{\delta}, 0\right)$, and a state representing immune activation, $S_L = (\bar{V}_p, \bar{e}, \bar{T}_p) = \left(\Omega, \frac{\pi\Omega}{\delta}, \frac{r_p}{\mu_p}\left(1 - \frac{\Omega}{K}\right)\right)$; where $\Omega = \frac{d\delta}{\alpha_p\delta - d\sigma\pi}$.

Proposition 2. *(1-3) exhibits the following dynamics.*

1. *S_0 is always unstable.*
2. *If $\Omega < 0$ or $\Omega > K$ then S_T is asymptotically stable and S_L does not exist.*
3. *If $0 < \Omega < K$ then S_T is unstable and S_L exists and is asymptotically stable.*

Proof. 1. and 2. The stability of S_0 and S_T follow from standard linearization techniques.
3. The proof of the stability of S_L is as follows.

$$J(\bar{V}_p, \bar{e}, \bar{T}_p)|_{S_L} = \begin{pmatrix} -\frac{r_p \bar{V}_p}{K} & 0 & -\mu_p \bar{V}_p \\ \pi & -\delta & 0 \\ \frac{\alpha_p \bar{T}_p}{1+\sigma\bar{e}} & -\frac{\alpha_p \sigma \bar{T}_p \bar{V}_p}{(1+\sigma\bar{e})^2} & 0 \end{pmatrix}.$$

The eigenvalues of J satisfy

$$\lambda^3 + a_1\lambda^2 + a_2\lambda + a_3 = 0,$$

where

$$\begin{aligned} a_1 &= \delta + \frac{r_p \bar{V}_p}{K}, \\ a_2 &= \frac{\delta r_p \bar{V}_p}{K} + \frac{\alpha_p \mu_p \bar{T}_p \bar{V}_p}{1+\sigma\bar{e}}, \\ a_3 &= \frac{\delta \alpha_p \mu_p \bar{T}_p \bar{V}_p}{(1+\sigma\bar{e})^2}. \end{aligned}$$

Clearly $a_1, a_2, a_3 > 0$ when S_L exists. Furthermore,

$$\begin{aligned} a_1 a_2 &= \left(\delta + \frac{r_p \bar{V}_p}{K}\right)\left(\frac{\delta r_p \bar{V}_p}{K} + \frac{\alpha_p \mu_p \bar{T}_p \bar{V}_p}{1+\sigma\bar{e}}\right) \\ &> \frac{\delta \alpha_p \mu_p \bar{T}_p \bar{V}_p}{1+\sigma\bar{e}} \\ &\ge \frac{\delta \alpha_p \mu_p \bar{T}_p \bar{V}_p}{(1+\sigma\bar{e})^2} = a_3. \end{aligned}$$

By Routh-Hurwitz criteria, we determine that S_L is locally asymptotically stable when it exists. □

Model analysis of mutation model

$$\frac{dV_p}{dt} = r_pV_p\left((1-\phi)-\frac{V_p+V_n}{K}\right)-\mu_pV_pT_p, \quad (4)$$
$$\frac{dV_n}{dt} = r_pV_n\left(1-\frac{V_p+V_n}{K}\right)+r_p\phi V_p-\mu_pV_nT_n, \quad (5)$$
$$\frac{de}{dt} = \pi V_p-\delta e, \quad (6)$$
$$\frac{dT_p}{dt} = \frac{\alpha_pV_pT_p}{1+\sigma e}-dT_p, \quad (7)$$
$$\frac{dT_n}{dt} = \frac{\alpha_nV_nT_n}{1+\sigma e}-dT_n, \quad (8)$$

The mutation model has several steady states. The first one is biologically irrelevant, $S_0=(0,0,0,0,0)$. The tolerance state of (4-8) is depicted by the absence of T-cell induced killing of V_n when V_p is lost completely, $S_{T_n}=(0,K,0,0,0)$.

There are four steady-states that represent immune activation. The first one represents immune activation against V_p but not V_n,

$$S_{L1}=(\bar{\vartheta}_1,\bar{\omega}_1,\bar{e}_1,\bar{\tau}_1,\bar{\sigma}_1)=\left(\Omega,\bar{\omega}_1,\frac{\pi}{\delta}\Omega,\bar{\tau}_1,0\right),$$

where

$$\frac{1}{K}\bar{\omega}_1^2 + \left(\frac{\Omega}{K}-1\right)\bar{\omega}_1-\Omega\phi=0, \quad (9)$$

and

$$\bar{\tau}_1 = \frac{r_p}{\mu_p}\left(1-\phi-\frac{\Omega+\bar{\omega}_1}{K}\right). \quad (10)$$

S_{L1} exists when $0<\Omega<K$ and $\phi<1-\frac{\Omega+\bar{\omega}_1}{K}$.

There are two steady states representing competent T-cell response to V_n but not V_p: one corresponding to small and intermediate percentage of V_p mutations,

$$S_{L2}=(\bar{\vartheta}_2,\bar{\omega}_2,\bar{e}_2,\bar{\tau}_2,\bar{\sigma}_2)=\left(\left(1-\phi-\frac{d}{K\alpha_n}\right)\frac{\alpha_n\delta K}{\alpha_n\delta+d\sigma\pi},(1-\phi)K-\bar{\vartheta}_2,\frac{\pi}{\delta}\bar{\vartheta}_2,0,\frac{r_p\phi}{\mu_p}\left(\frac{\bar{\vartheta}_2}{\bar{\omega}_2}+1\right)\right)$$

which exists when $\phi<1-\frac{d}{K\alpha_n}$; and one corresponding to large percentage of mutations leading to complete removal of V_p,

$$S_{L3}=(\bar{\vartheta}_3,\bar{\omega}_3,\bar{e}_3,\bar{\tau}_3,\bar{\sigma}_3)=\left(0,\frac{d}{\alpha_n},0,0,\frac{r_p}{\mu_p}\left(1-\frac{d}{\alpha_nK}\right)\right),$$

which exists when $K>\frac{d}{\alpha_n}$.
The last steady state corresponds to T-cells response to both viruses types,

$$S_{L4}=(\bar{\vartheta}_4,\bar{\omega}_4,\bar{e}_4,\bar{\tau}_4,\bar{\sigma}_4)=\left(\Omega,\frac{\alpha_p}{\alpha_n}\Omega,\frac{\pi}{\delta}\Omega,\bar{\tau}_4,\bar{\sigma}_4\right),$$

where

$$
\begin{aligned}
\bar{\tau}_4 &= \frac{r_p}{\mu_p}\left(1-\phi-\frac{\alpha_p+\alpha_n}{\alpha_n}\frac{\Omega}{K}\right), && (11)\\
\bar{\sigma}_4 &= \frac{r_p}{\mu_p}\left(1+\phi\frac{\alpha_n}{\alpha_p}-\frac{\alpha_p+\alpha_n}{\alpha_n}\frac{\Omega}{K}\right). && (12)\\
& && (13)
\end{aligned}
$$

This state exist if $\frac{\alpha_p}{\alpha_n}\left(\frac{\alpha_p+\alpha_n}{\alpha_n}\frac{\Omega}{K}-1\right)<\phi<1-\frac{\alpha_p+\alpha_n}{\alpha_n}\frac{\Omega}{K}$. Note that the inequality fails for $\Omega>K$. Therefore, S_{L4} doest not exist when $0>\Omega>K$.

Proposition 3. *When $K<d/\alpha_n$ the steady state S_{T_n} is locally asymptotically stable and S_{L2}, S_{L3} do not exist.*

Proof. The stability results follow from standard linearization techniques. □

Proposition 4. *If $\Omega=\frac{d\delta}{\alpha_p\delta-d\pi\sigma}>K$, then S_T is locally asymptotically stable in the system (1-3) and S_{T_n}, S_{L1} and S_{L4} do not exist in the system (4-8).*

Proof. This result follows from Proposition 2 and the existence of S_T and S_{L1}. □

Proposition 5. *If $1-\frac{\Omega}{K}\left(1+\frac{\alpha_p}{\alpha_n}\right)<\phi<1-\frac{d}{K\alpha_n}$, S_{L2} is locally asymptotically stable.*

Proof. The Jacobian for S_{L2} is

$$
J_{S_{L2}}=\begin{bmatrix}
-r_p\frac{\bar{\vartheta}_2}{K} & -r_p\frac{\bar{\vartheta}_2}{K} & 0 & -\mu_p\bar{\vartheta}_2 & 0\\
r_p\phi-r_p\frac{\bar{\omega}_2}{K} & -r_p\phi\frac{\bar{\vartheta}_2}{\bar{\omega}_2}-r_p\frac{\bar{\omega}_2}{K} & 0 & 0 & -\mu_p\bar{\omega}_2\\
\pi & 0 & -\delta & 0 & 0\\
0 & 0 & 0 & \frac{\alpha_p\bar{\vartheta}_2}{1+\sigma\bar{e}_2}-d & 0\\
0 & \frac{\alpha_n\bar{\sigma}_2}{1+\sigma\bar{e}_2} & -\alpha_n\sigma\frac{\bar{\omega}_2\bar{\sigma}_2}{(1+\sigma\bar{e}_2)^2} & 0 & 0
\end{bmatrix}.
$$

Notice that $\lambda_1=\frac{\alpha_p\bar{\vartheta}_2}{1+\sigma\bar{e}_2}-d=\left(\frac{\bar{\vartheta}_2}{\Omega}-1\right)d$. If $\phi>1-\frac{\Omega}{K}\left(1+\frac{\alpha_p}{\alpha_n}\right)$ then $\lambda_1<0$. $\lambda_{2,3,4,5}$ solve the following polynomial.

$$
\begin{aligned}
\lambda^4 &+ A\lambda^3+B\lambda^2+C\lambda+D=0,\\
A &= r_p\phi\frac{\bar{\vartheta}_2}{\bar{\omega}_2}+r_p(1-\phi)+\delta,\\
B &= r_p\delta(1-\phi)+r_p^2\phi(1-\phi)\frac{\bar{\vartheta}_2}{\bar{\omega}_2}+r_pd\phi(1+\frac{\bar{\vartheta}_2}{\bar{\omega}_2})+r_p\phi\delta\frac{\bar{\vartheta}_2}{\bar{\omega}_2},\\
C &= r_p\delta d\phi(1+\frac{\bar{\vartheta}_2}{\bar{\omega}_2})+r_p^2\phi(1-\phi)(d+\delta)\frac{\bar{\vartheta}_2}{\bar{\omega}_2},\\
D &= r_p^2d\phi(\bar{\vartheta}_2+\bar{\omega}_2)\bar{\vartheta}_2\frac{\alpha_n\delta+d\sigma\pi}{\alpha_n\bar{\omega}_2K}.
\end{aligned}
$$

Since $A>0$, $D>0$, $AB-C>0$, and $C(AB-C)-A^2D>0$ by Routh Hurwitz condition we have that the polynomial has solutions with negative real parts. Therefore, when $1-\frac{\Omega}{K}\left(1+\frac{\alpha_p}{\alpha_n}\right)<\phi<1-\frac{d}{K\alpha_n}$, S_{L2} is locally asymptotically stable. □

Proposition 6. *If $\phi>1-\frac{d}{K\alpha_n}$ and $\frac{d}{\alpha_n}<K$, S_{L3} is locally asymptotically stable.*

Proof. The Jacobian for S_{L3} is

$$J_{S_{L3}} = \begin{bmatrix} r_p(1-\phi) - \frac{r_p}{K}\frac{d}{\alpha_n} & 0 & 0 & 0 & 0 \\ r_p\phi - \frac{r_p}{K}\frac{d}{\alpha_n} & -\frac{r_p}{K}\frac{d}{\alpha_n} & 0 & 0 & -\mu_p\frac{d}{\alpha_n} \\ \pi & 0 & -\delta & 0 & 0 \\ 0 & 0 & 0 & -d & 0 \\ 0 & \frac{r_p\alpha_n}{\mu_p}\left(1-\frac{d}{K\alpha_n}\right) & -\frac{\sigma d r_p}{\mu_p}\left(1-\frac{d}{K\alpha_n}\right) & 0 & 0 \end{bmatrix}.$$

Notice that $\lambda_1 = r_p(1-\phi) - \frac{r_p}{K}\frac{d}{\alpha_n}$, $\lambda_2 = -\delta$, and $\lambda_3 = -d$. When $\phi > 1 - \frac{d}{K\alpha_n}$ and $\frac{d}{\alpha_n} < K$, $\lambda_1 < 0$. $\lambda_{4,5}$ are the eigenvalues of

$$\begin{pmatrix} -\frac{r_p}{K}\frac{d}{\alpha_n} & -\frac{d\mu_p}{\alpha_n} \\ \frac{r_p\alpha_n}{\mu_p}\left(1-\frac{d}{K\alpha_n}\right) & 0 \end{pmatrix}.$$

Since

$$\begin{aligned} \text{tr} &= -\frac{r_p}{K}\frac{d}{\alpha_n} < 0, \\ \det &= r_p d\left(1-\frac{d}{K\alpha_n}\right) > 0, \end{aligned}$$

when $K\alpha_n > d$, the eigenvalues $\lambda_{4,5}$ are always negative. □

References
